# Colony-Forming Efficiency Assay to Assess Nanotoxicity of Graphene Nanomaterials

**DOI:** 10.3390/toxics10050236

**Published:** 2022-05-05

**Authors:** Hansol Won, Sung-Hyun Kim, Jun-Young Yang, Kikyung Jung, Jayoung Jeong, Jae-Ho Oh, Jin-Hee Lee

**Affiliations:** Division of Toxicological Research, National Institute of Food and Drug Safety Evaluation, Ministry of Food and Drug Safety, 187, Osongsaengmyeong 2-Ro, Cheongju 28159, Korea; hanssol2@korea.kr (H.W.); tjdgus32@kakao.com (S.-H.K.); yangjy@korea.kr (J.-Y.Y.); kikyung@korea.kr (K.J.); 0jjy@korea.kr (J.J.); chopin68@korea.kr (J.-H.O.)

**Keywords:** graphene, cytotoxicity, CFE, interference

## Abstract

The nano-market has grown rapidly over the past decades and a wide variety of products are now being manufactured, including those for biomedical applications. Despite the widespread use of nanomaterials in various industries, safety and health effects on humans are still controversial, and testing methods for nanotoxicity have not yet been clearly established. Nanomaterials have been reported to interfere with conventional cytotoxicity tests due to their unique properties, such as light absorption or light scattering. In this regard, the colony-forming efficacy (CFE) assay has been suggested as a suitable test method for testing some nanomaterials without these color-interferences. In this study, we selected two types of GNPs (Graphene nanoplatelets) as test nanomaterials and evaluated CFE assay to assess the cytotoxicity of GNPs. Moreover, for further investigation, including expansion into other cell types, GNPs were evaluated by the conventional cytotoxicity tests including the 3-(4,5-dimethylthiazol-2-yl)-5-(3-carboxymethoxyphenyl)-2-(4-sulfophenyl)-2H-tetrazolium (MTS), Cell Counting Kit-8 (CCK-8), and Neutral red uptake (NRU) assay using MDCK, A549 and HepG2 cells. The results of CFE assay suggest that this test method for three cell lines can be applied for GNPs. In addition, the CFE assay was able to evaluate cytotoxicity regardless more accurately of color interference caused by residual nanomaterials.

## 1. Introduction

In recent years, due to the rapid growth of nanotechnology, various manufacturing nanomaterials are being produced, and nanomaterials are used in various industries such as batteries, electrodes, cosmetics, displays and biomedical engineering [1,2,3]. The growth of the nano-industry affects our lives in a more prosperous manner and contributes to it by providing various benefits, however like a ‘double-edged sword’ it has the potential to induce human toxicity, both large and small when exposed to the body. Therefore, it is crucial to develop an accurate nanotoxicity evaluation method to understand the toxicity of these nanomaterials.

The Organization for Economic Cooperation and Development (OECD), European Union (EU) and other organizations stipulate the following for manufactured nanomaterials: ‘Materials with a size less than 100 nm made for this purpose’ [4,5]. As such, nanomaterials are nanoscopic in size, and the risk of nanomaterial products stem from its small size and the unique physicochemical properties of the nanomaterial. Taking the physical ‘shape’ as an example, carbon nanotube (CNT) nanomaterials with acicular structures such as asbestos or glass fibers have risks such as cancer-causing potential [6,7]. In addition, high surface reaction power and surface charge, due to their very small size, can contribute to allowing nanomaterials to be easily grouped and accumulated into cells [8,9].

Unlike general chemicals, nanomaterials have unique properties that make them nearly non-soluble; thus, during measurements, solid nanomaterials are detected in particular areas such as the bottom of a well, deposition on cell membranes and during intracellular uptake, which can interfere with the system and generate unreliable data [10]. Guadagnini et al. [11] also reported significant differences observed in nanomaterials interference for cytotoxicity analysis depends on the nature of nanomaterials. These problems can make it difficult to determine the exact cytotoxicity of nanomaterials, so a solution to the effects of nanomaterial interference is needed.

Colony forming efficiency (CFE) assay used to measure cellular ability to form colonies was described in OECD detailed review paper and Joint Research Centre (JRC) reports [12,13]. This in vitro assay can be used to determine cytotoxicity induced by nanomaterials. It can be performed with any adherent cells that are able to form colonies including human adult low calcium high temperature (HaCaT), Madin Darby canine kidney cell line (MDCK), human lung cancer cells (A549), human liver carcinoma cell line (HepG2) and immortalized mouse fibroblast cell line (Balb/3T3) cells. The great advantage of CFE assay is a label-free test that reduces the possibility of the incident of nanomaterials interferences. This testing method calculates cell viability by comparing the number of colonies in the vehicle control after treatment with toxicants.

We conducted a test to analyze the colony formation efficacy of various nanomaterials in MDCK cells through the OECD-JRC report [13]. In the present study, we tried to evaluate the CFE assay method for GNPs, a kind of carbon nanomaterial. In addition, we want to establish a GNP’s CFE assay protocol based on A549 and HepG2 cells, which is different from the CFE conditions of MDCK cells proposed by the OECD.

In this study, the applicability of carbon-based graphene nanomaterials to a total of three cell lines was evaluated through CFE assay, an in vitro test method independent of the effect of color interference. Also, we aimed to compare the cytotoxicity assay of the conventional colorimetric cytotoxicity assay with that of the CFE assay.

## 2. Materials and Methods

### 2.1. Graphene Nanomaterials

Two types of GNPs (product No. 06-0225, product No. 06-0230) materials were purchased from Strem Chemicals (Newburyport, MA, USA). Their morphological images were confirmed by transmission electron microscopy (TEM; JEM-1200EX II, JEOL, Tokyo, Japan). The zeta potentials of the GNPs were measured using a Zetasizer Nano ZS instrument (Malvern Instruments, Malvern Hills, UK). To confirm the unique physicochemical properties of GNPs, this assay was measured in PBS and culture medium (DMEM contained with 10% FBS). In order to evaluate the dispersion stability of the test substance, the final working time was measured up to 72 h. To evaluate the dispersion stability of GNPs, a dynamic light scattering (DLS) was measured using a Zetasizer (Malvern). The levels of endotoxin were evaluated using an Endpoint Chromogenic Limulus Amoebocyte Lysate (LAL) QCL-1000 assay (Cambrex, Walkersville, MD, USA). Endotoxins were measured according to the protocols provided by the kit’s manufacturer.

### 2.2. Preparation of Nanomaterials Suspensions

GNP’s suspension was prepared by slightly modifying described methods [14,15]. Briefly, the GNPs stock (10× fold) solutions were dispersed in PBS and sonicated at 40 kHz with 100 W output power for 30 min in an ultra-sonicator (Saehan-Sonic, Seoul, South Korea). Thereafter, Dulbecco’s Modified Eagle’s Medium (DMEM) (Life Technologies, Grand Island, NY, USA) supplemented with 10% fetal bovine serum (FBS) (Life Technologies), 100 U/mL penicillin (Life Technologies), and 100 μg/mL streptomycin (Life Technologies) was added to different working concentrations (Table 1). Initially, the concentration of GNPs was set based on a JRC report, which was previously evaluated by reference to the concentration of the same single-wall carbon nanotubes (swCNTs) [13]. The concentrations of two GNPs were finally set by performing a preliminary toxicity assessment based on the concentration of the above carbon nanotubes and up-adjusting the test concentration according to the results.

### 2.3. Cell Culture

MDCK (product No. CCL-34), A549 (product No. CCL-185), and HepG2 (product No. HB-8065) cell lines were purchased from American Type Culture Collection (ATCC; Manassas, VA, USA). The cells were cultured in DMEM medium supplemented with 10% FBS, 100 U/mL penicillin and 100 μg/mL streptomycin. Three types of cells were sub-cultured every 2–4 days at about 80% confluence. For the experiments, cell density was adjusted according to the conditions of each cytotoxicity method and seeded on the 96 well culture plates or 60 × 15-mm Petri dish. Then, culture medium was replaced with a fresh medium and incubated in a humidified atmosphere condition of 5% CO_2_ at 37 °C.

### 2.4. Colony Forming Efficiency Assay Methods

CFE assay was performed as previously described to study the cytotoxicity induced by two types of GNPs [13]. The cells were seeded at a density of 200 cells/dish (MDCK) in 3 mL complete culture medium at least in three replicates for each treatment. In the same procedure, 400 cells/dish for A549 cells and 200 cells/dish for HepG2 cells were inoculated, respectively. After 24 h, the treatment suspensions of nanomaterials and positive control (sodium chromate, Na_2_CrO_4_, product No. 307831, Sigma-Aldrich, St. Louis, MO, USA) were added to the cells. After 72 h of exposure, the medium was changed with a fresh complete culture medium. Considering the growth cycle of each cell line colony, after 5 days (MDCK), 8 days (HepG2) and 10 days (A549), each cell was fixed for 20 min with 3.7% (*v*/*v*) of formaldehyde solution (Sigma-Aldrich) in PBS without calcium, magnesium and sodium bicarbonate (Life Technologies, product No. 14190-250), and stained for 30 min with 0.4% (v/v) Giemsa solution (Sigma-Aldrich, product No. GS500) in ultrapure water. Colonies were manually scored under a stereomicroscope. The results were expressed as CFE (%) = [(average of treatment colonies/average of control colonies) × 100] and the corresponding standard error means [SEM% = SD/√ (number of treatments)].

(1) $$\mathrm{Colony}\;\mathrm{Forming}\;\mathrm{Efficiency}\;\left(\%\right)=\frac{\mathrm{Average}\;\mathrm{of}\;\mathrm{treatment}\;\mathrm{colonies}\times 100}{\mathrm{Average}\;\mathrm{of}\;\mathrm{control}\;\mathrm{colonies}}$$

### 2.5. Cytotoxicity Measurement of Colorimetric Based Assay

To confirm the difference between the existing tests measuring cytotoxicity of the test substance and the CFE assay, two types of GNPs were evaluated using three commonly used colorimetric cytotoxicity assays. The MTS assay; each cell was seeded at 3 × 10^4^ cells/well in 96-well plates and cultured overnight and incubated for another 24 h. The conversion of MTS tetrazolium salt into its reduced formazan form was assessed with the CellTiter 96 AQueous Non-Radioactive Cell Proliferation Assay kit (Promega, Madison, WI, USA) following the manufacturer’s protocol. The absorbance was read at 450 nm on a Synergy HT Multimode Microplate Reader (Bio-Tek Instruments, Winooski, VT, USA).

Cell Counting Kit-8 (CCK-8) assay; to evaluate the cell viability, cells were seeded into 96-well plates at a density of 1 ×10^4^ cells/mL and incubated overnight to reach approximately 80% confluence. Followed by the addition of suspension containing either nanomaterials or positive control and then were incubated for 24 h at each different dose. After 24 h, the cell viability was measured using a CCK-8 assay kit (Dojindo Molecular Technologies, Gaithersburg, MD, USA). 

Neutral red uptake (NRU) assay is a dye exclusion assay (Sigma-Aldrich, Cat #N2889). Briefly, each cell was seeded at 5 × 10^3^ cells/well in 96-well plates for 24 h prior to the treatments. After nanomaterials suspension treatment, plates were incubated for 2 h with a supplemented medium containing 40 μg/mL of neutral red. Cells were subsequently washed twice with Dulbecco’s Phosphate Buffered Saline (DPBS) and the dye was extracted with 200 μL destaining solution (ethanol, deionized water, and glacial acetic acid, 50:49:1 *v*/*v*). The absorbance was read at 540 nm using a microplate reader. Cell viability in terms of percentage of control was expressed in the same manner as for the MTS assay. All four types of test result data were expressed as mean ± SEM (*n* = 3) using GraphPad Prism V5.0 (GraphPad Software, San Diego, CA, USA). 

## 3. Results

### 3.1. Physicochemical Characteristics of Graphene Nanomaterials

**Figure 1:**
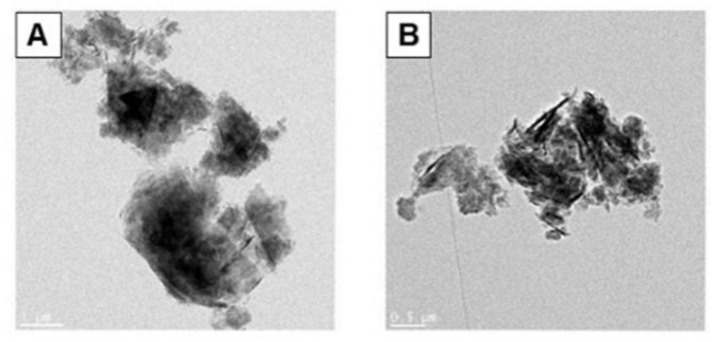
Morphological images of differential graphene nanomaterials observed by transmission electron microscopy. (**A**) GNPs-1 (300 m^2^/g, bar = 1 μm) and (**B**) GNPs-2 (500 m^2^/g, bar = 0.5 μm).

The morphological characteristics of graphene nanomaterials were confirmed through transmission electron microscopy (TEM) images (Figure 1). TEM images of graphene nanoplatelets (GNPs)-1 and GNPs-2 could not measure the average diameter, but according to the information provided by the manufacturer, the average diameter was <2 μm, and thickness was a few nanometers. Measurement of the zeta potential showed that two GNPs were negatively charged, with charge in phosphate buffered saline (PBS) or working solution. As a result of the dispersion stability measurement, the two substances showed a similar size distribution up to 72 h (Table 2). There was no statistical significance. Through the results of Limulus Amoebocyte Lysate (LAL) test, it was confirmed that all nanomaterials did not show contamination by endotoxin.

### 3.2. Cytotoxicity Evaluation of GNPs Using CFE Assay for Selected Cell Lines

**Figure 2:**
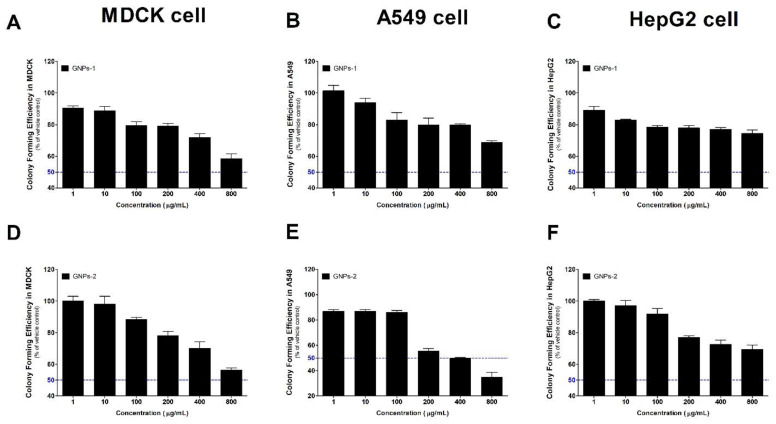
The colony-forming efficacy (CFE) assay results of graphene nanomaterials to MDCK, A549, and HepG2 cells. Results of GNPs-1 in (**A**) MDCK, (**B**) A549 and (**C**) HepG2 cells. Results of GNPs-2 in (**D**) MDCK, (**E**) A549, (**F**) HepG2 cells. Data are expressed as mean ± SEM (*n* = 3). Blue line; 50 = The half maximal.

The cytotoxicity of GNPs was evaluated by performing a CFE assay using three types of cell lines. A graph of the calculated CFE compared to the vehicle control group was presented in Figure 2 and used CFE dish images for each cell line was presented in Supplementary Materials (Figures S1–S3). First, the result of the colony forming ability test using MDCK cells at a concentration of 200 cells/dish was able to obtain cytotoxicity results in a dose-dependent manner in GNPs-1 and GNPs-2. It was confirmed that A549 cells with a concentration of 400 cells/dish, which are additional cell lines other than MDCK, were capable of optimal colony count at 10 days, and optimized at eight days of 200 cells/dish in HepG2 cells. Like the MDCK results, the results of colony formation tests using two cell lines confirmed that the cytotoxicity of nanomaterials was identified in a dose-dependent manner.

### 3.3. Differences of Cytotoxicity between Colorimetric Assays

**Figure 3:**
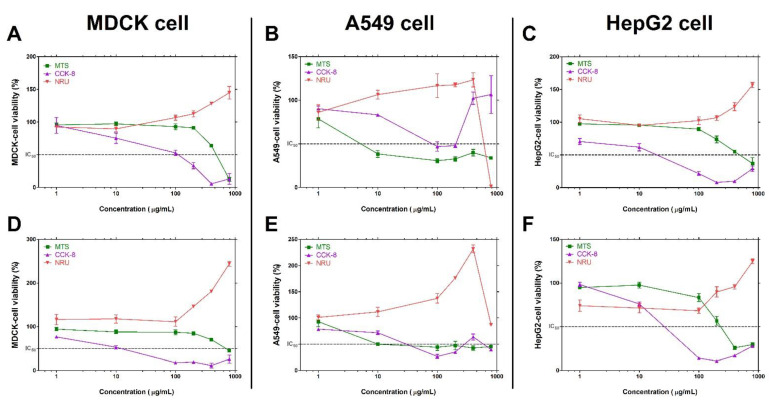
The comparison with results of colorimetric cytotoxicity assays treated with graphene nanomaterials. The cytotoxicity results of GNPs-1 in (**A**) MDCK, (**B**) A549 and (**C**) HepG2 cells. The cytotoxicity results of GNPs-2 in (**D**) MDCK, (**E**) A549, (**F**) HepG2 cells. Data are expressed as mean ± SEM (*n* = 3).

The in vitro cytotoxic effect of graphene nanomaterials was explored against MDCK, A549 and HepG2 cells in comparison with CFE assay and colorimetric cytotoxicity assay (Figure 3). Some data of the neutral red uptake (NRU) assay (red line) in the three cells showed cell viability higher than the viability value of the control group. In other words, it was observed that the cell viability rather increased as the concentration of graphene increased. In the cell counting kit-8 (CCK-8) assay results (purple line), a concentration-dependent cytotoxicity trend was observed at relatively low concentrations, but an increase-pattern in survival rate was observed with increasing optical density (OD) values at high concentrations. Especially, in A549 cells of GNPs-1 in Figure 3B, IC50 was observed at low-concentration, however cell viability showed a tendency to increase to more than 100% at high concentration. In the results of the 3-(4,5-dimethylthiazol-2-yl)-5-(3-carboxymethoxyphenyl)-2-(4-sulfophenyl)-2H-tetrazolium (MTS) assay (green line), the formazan measurement method based on mitochondrial activity, it showed a trend of concentration-dependent cell viability more sensitively than the other two methods. However, it appeared that cell viability increased when the OD value was increased at some high concentrations as shown in Figure 3B,F.

## 4. Discussion

Although the advantages of various nanomaterials enrich our society, it is essential to accurately evaluate the toxicity of nanomaterials to ensure the safety of workers in plants manufacturing the materials and consumers who consume products. Therefore, there is a need to develop a reliable toxicity analysis method for nanomaterials. Recently, the toxicity of nanomaterials has been identified and reported through various studies, and based on these research results, the OECD has revised the animal test guidelines such as inhalation toxicity evaluation prepared based on chemical substances by reflecting the content of nanomaterials.

Animal testing is also important in evaluating the toxicity of nanomaterials, but in vitro testing is a test method that may be appropriate for screening the toxicity of nanomaterials to various target organs under controlled conditions that cannot be performed in vivo testing. Oberdorster et al. propose a portal-of-entry toxicity test for target organ toxicity such as lung, skin, endothelium, liver and kidney as an in vitro technique [16]. The OECD-JRC report exemplifies some test cases of nanomaterials, and a preemptive study was conducted [13]. Through this work, the OECD established and proposed a “test protocol” for a colony-forming efficacy assay based on MDCK cells. However, since the test method was verified only for MDCK cells, research was needed to apply it to various cells. To utilize the colony test for a variety of cell lines, Ponti et al. reported studies applying various adherent cells such as Caco-2, HepG2, etc [17]. In our study, we established the applicability of the CFE assay using the alveolar epithelial cell line A549 and the hepatocyte cell line HepG2 cells as organ-derived cell lines closely related to the exposure and accumulation of nanomaterials. Based on the test results, different time-points are required to prepare a colony optimized for cells, so there is a limitation that a ‘standardized number of cells’ cannot be specified. However, in the end, it was confirmed that these cell lines could be used in the CFE test.

Other studies involving the testing of graphene cytotoxicity with CFE assay in vitro [18,19,20]. Our study performs validation of cell line expansion of swCNTs (Figure S4), and also reports the CFE evaluation results of carbon-based graphene nanomaterials that have not been reported in previous studies [13]. GNP is a type of carbon nanomaterial, which has recently attracted great attention in various fields including biomedical [21]. In general, as well known, nanomaterials were insoluble in almost solvents, and most readily form agglomerates as confirmed by transmission electron microscopy (Figure 1) [22]. Since the nanotoxicity can be accurately evaluated only when the nanomaterials used are uniformly dispersed, we tried to find a dispersion method optimized for nanomaterials. According to the papers reported on dispersion, ‘serum protein’ is known as a very useful dispersing agent [23,24]. In fact, it has been reported that the large aggregation of nanomaterials in a solvent was reduced by gentle aggregation in the final solution after dispersion using serum [25,26]. Therefore, by applying this method, the protein-corona coating operation was performed using FBS on the nanomaterial stock solution to induce the most homogeneous dispersion of the particles. In addition, since the mechanical dispersion operation of the ultrasonic disperser can contribute to the homogenization of the test material, the operation was additionally reflected [23].

The contrasting difference between the CFE assay and the conventional cytotoxicity assay for GNPs substances may suggest that the CFE assay was a reliable in vitro toxicity assay for GNPs [10,11,27]. Nanomaterials are either absorbed by cells or deposited on cell membranes or wells. These particles interact with cells or remain in culture plate wells despite multiple washing operations [17,28]. Traditional colorimetric-cytotoxicity assays use absorbance to evaluate the toxicity of test materials by calculating the OD values of the control and test groups and calculating the cytotoxicity in ‘percentage (%)’ [29,30,31,32]. If conventional absorbance-based colorimetric measurements were performed in the presence of such residual nanomaterials, incorrect results may be obtained through distortion of OD values. According to Guadagnini et al. (2015) [11], TiO_2_ nanoparticles can cause false-negative results because they have the property of increasing absorbance when measured by a colorimetric method. Moreover, Wörle-Knirsch et al., Casey et al., Monteiro-Riviere et al. reported that the evaluation of carbon-based nanomaterials, which was also used in our study, may not be suitable for cytotoxicity evaluation due to color interference of the material [33,34,35]. As such, absorbance-based tests using nanomaterials are highly likely to cause distortion of results, such as increased cell viability in the presence of color interference. Additionally, if strong washing is performed to remove the remaining nanomaterials, there is a possibility that it may cause loss of cells attached to the bottom of the well and lower the viability of the original cells.

In order to avoid distortion of the measurement result due to residual nanomaterials, a method of transferring the supernatant to a new plate may be considered [36]. When this method is applied, the uptake state in the cell or the substances strongly attached to the outer membrane are excluded from the absorbance measurement so that distortion is not induced. However, in the case of nanomaterials that dissolve rapidly and release metal ions, it can affect the color of the medium and induce distortion in the supernatant itself. For example, in the case of CuO nanoparticles, Cu ions chemically inactivate the intracellular formazan formation cascade in LDH analysis, which is one of the color-metric assays, resulting in false-negative results [26]. Therefore, the CFE assay is a label-free assay that counts the number of colonies in evaluating these kinds of nanomaterials that can affect the supernatant itself, so it can be a good alternative in vitro assay [37,38].

In conclusion, we reported CFE results using three types of cells for (GNPs, which have not been evaluated so far. This study successfully established applicability by applying the GNP’s CFE assay to MDCK, A549 and HepG2 cells. Of course, nanomaterials have different shapes, sizes, colors, etc., so the appropriate test methods may be different, respectively. Because our study applied only two GNPs, there may be some limitations. Therefore, it is thought that more data accumulation of nanomaterials for the CFE test method is needed.

## 5. Conclusions

In this study, the toxicity evaluation of nano-graphene in three cell lines was successfully confirmed, and the optimal time zone was confirmed for each cell line. Currently, the CFE test method of nanomaterials is being prepared for OECD guidelines, so it is judged that these cell-specific established model studies can contribute to international standards or guidelines. However, further investigation is needed because a better understanding of the toxicity of these NP requires more information about immune activity and ROS generation potential.

## Figures and Tables

**Table 1:** Test concentration of the two different carbon nanomaterials.

Nanomaterials	CAS RN	Test Concentration * (μg/mL)
GNPs-1 (300 m^2^/g)	7782-42-5	1, 10, 100, 200, 400, 800
GNPs-2 (500 m^2^/g)	7782-42-5	1, 10, 100, 200, 400, 800

* The concentration of GNPs was set based on a JRC report [13]. GNPs = graphene nanoplatelets.

**Table 2:** Physicochemical characterization of the graphene nanomaterials.

Characterization	GNPs-1	GNPs-2
Average diameter (nm) *	<2 μm	<2 μm
	(a thickness of a few nanometers)	
Surface area (m^2^/g) *	300	500
Zeta potential (mV) in PBS **	−30.01 ± 4.30	−33.32 ± 4.91
Zeta potential (mV) in DMEM **	−26.82 ± 0.69	−25.78 ± 0.81
Endotoxin (EU/mL)	<0.1	<0.1
Dispersion stability measurement using DLS (nm) **		
0 h	3018.00 ± 213.55	2929.00 ± 66.47
24 h	3116.67 ± 684.92	3074.50 ± 303.35
72 h	3416.00 ± 823.07	3467.00 ± 934.80
pH		
In DMEM	10.81	
In working solution **	8.87	8.56

Data are expressed as mean ± standard error of the mean (*n* = 6). * This data used material information provided by the manufacturer. ** Working concentration was 800 μg/mL (Measurement was performed by diluting × 100 fold in DW at the highest concentration). GNPs = graphene nanoplatelets, PBS = phosphate buffered saline, DMEM = Dulbecco’s modified Eagle’s medium, EU = endotoxin unit, DLS = Dynamic light scattering.

## Data Availability

The original contributions presented in the study are included in the article/Supplementary Material, further inquiries can be directed to the corresponding authors.

## Supplementary Materials

The following are available online at https://www.mdpi.com/article/10.3390/toxics10050236/s1, Figure S1: The culture dish images for Colony Forming Efficiency (CFE) evaluation of MDCK cell line treated with graphene nanomaterials, Figure S2: The culture dish images for Colony Forming Efficiency (CFE) evaluation of A549 cell line treated with graphene nanomaterials, and Figure S3: The culture dish images for Colony Forming Efficiency (CFE) evaluation of HepG2 cell line treated with graphene nanomaterials, Figure S4: The validation data for Colony Forming Efficiency (CFE) evaluation of (A) MDCK, (B) A549, and (C) HepG2 cell line treated with single wall carbon nanotubes (swCNTs).
