# Supplementary material for: Colony-Forming Efficiency Assay to Assess Nanotoxicity of Graphene Nanomaterials
Source: Toxics. 2022 May 5;10(5):236. doi: 10.3390/toxics10050236 (PMC9146674; doi:10.3390/toxics10050236)
Supplement: Supplementary file 1 [file toxics-10-00236-s001.zip › toxics-1689586-supplementary.pdf]

toxics

Supplementary Materials: Colony-Forming Efficiency Assay to Assess Nanotoxicity of Graphene Nanomaterials

Hansol Won, Sung-Hyun Kim, Jun-Young Yang, Kikyung Jung, Jayoung Jeong, Jae-Ho Oh and Jin-Hee Lee

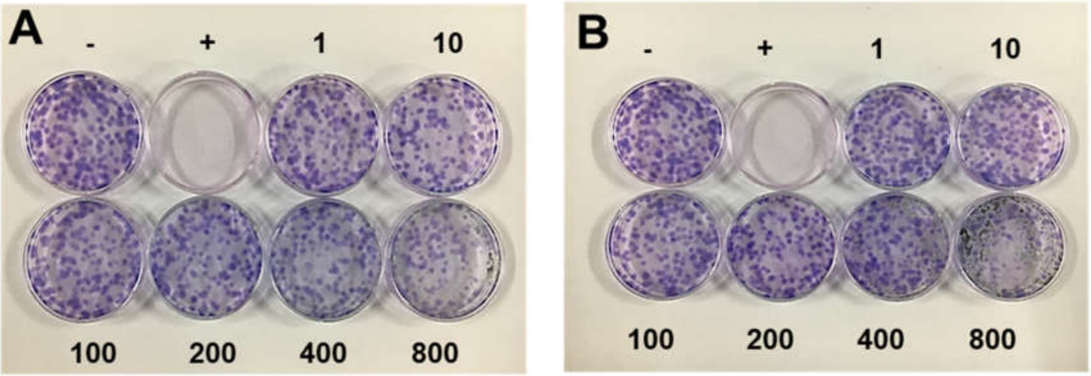

Figure S1. The culture dish images for Colony Forming Efficiency (CFE) evaluation of MDCK cell line treated with graphene nanomaterials. The captured image was taken using the OLYMPUS CAMEDIA C-4000 Zoom Digital Camera (Olympus Optical Company, Japan) (**A**) GNPs-1 (300 m2/g) and (**B**) GNPs-2 (500 m2/g).

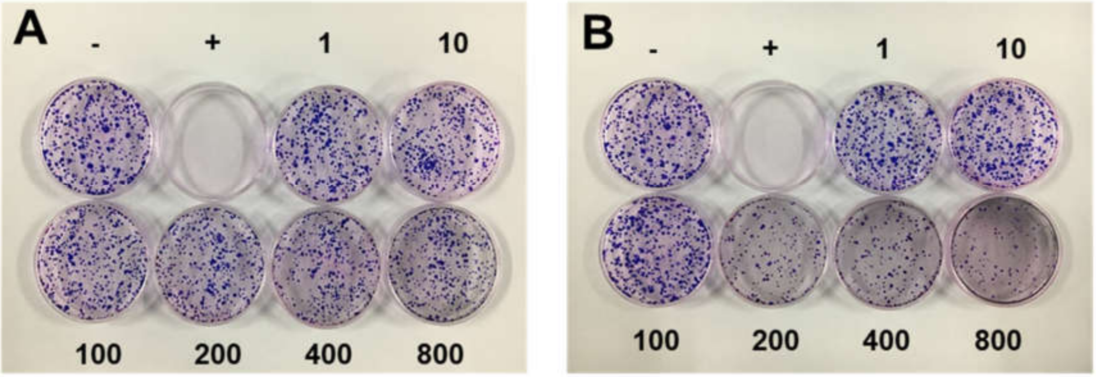

Figure S2. The culture dish images for Colony Forming Efficiency (CFE) evaluation of A549 cell line treated with graphene nanomaterials. The captured image was taken using the OLYMPUS CAMEDIA C-4000 Zoom Digital Camera (Olympus Optical Company, Japan) (**A**) GNPs-1 (300 m2/g) and (**B**) GNPs-2 (500 m2/g).

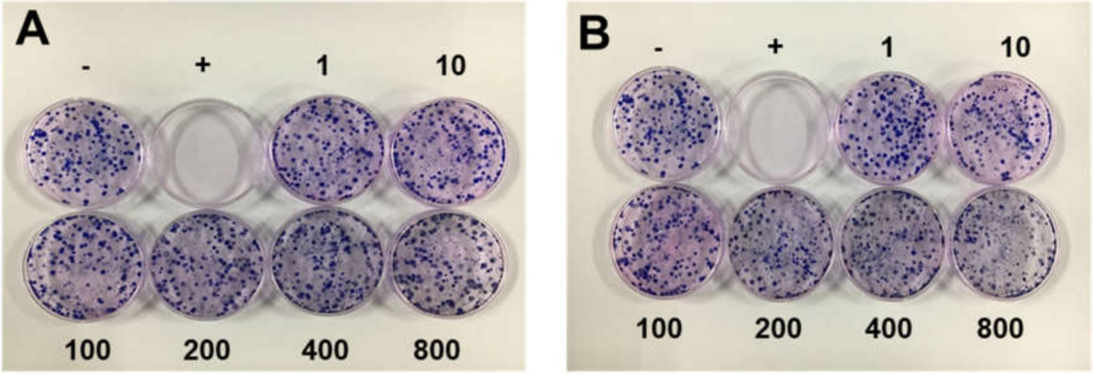

Figure S3. The culture dish images for Colony Forming Efficiency (CFE) evaluation of HepG2 cell line treated with graphene nanomaterials. The captured image was taken using the OLYMPUS CAMEDIA C-4000 Zoom Digital Camera (Olympus Optical Company, Japan) (**A**) GNPs-1 (300 m2/g) and (**B**) GNPs-2 (500 m2/g).

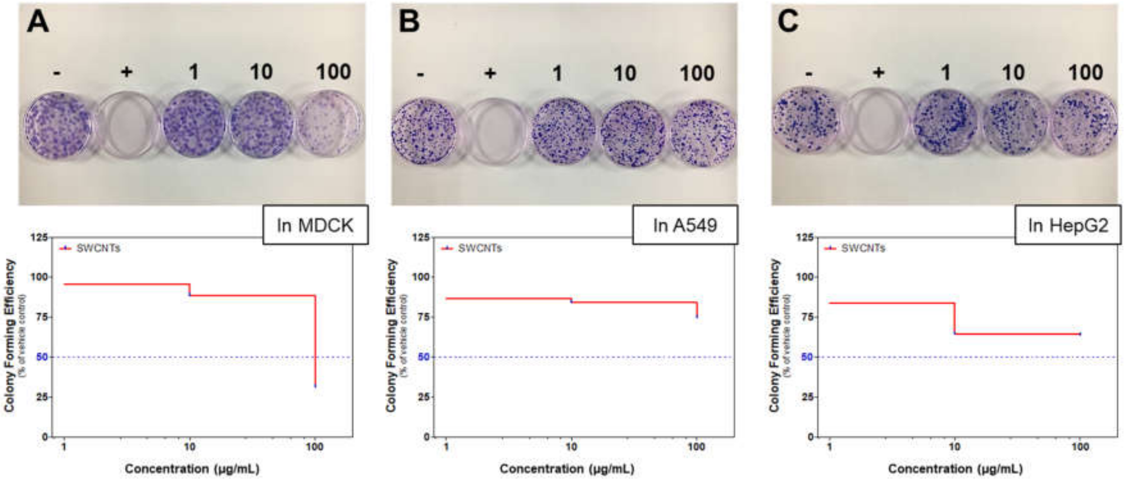

Figure S4. The validation data for Colony Forming Efficiency (CFE) evaluation of (**A**) MDCK, (**B**) A549, and (**C**) HepG2 cell line treated with single wall carbon nanotubes (swCNTs). Data are expressed as percent of survival mean values (n = 3). The captured image was taken using the OLYMPUS CAMEDIA C-4000 Zoom Digital Camera (Olympus Optical Company, Japan). Blue line; 50 = The half maximal inhibitory concentration (IC50).
